# Family history tools for primary care: A systematic review

**DOI:** 10.1080/13814788.2022.2061457

**Published:** 2022-05-05

**Authors:** Špela Miroševič, Zalika Klemenc-Ketiš, Borut Peterlin

**Affiliations:** a Department of Family Medicine, Medical Faculty Ljubljana, Ljubljana, Slovenia; b Department of Family Medicine, Faculty of Medicine, University of Maribor, Maribor, Slovenia; c Community Health Centre Ljubljana, Ljubljana, Slovenia; d Clinical Institute for Medical Genetics, University Medical Centre Ljubljana, Ljubljana, Slovenia

**Keywords:** Genetics, incl. family history, general practice/family medicine, general, systematic reviews, meta-analyses

## Abstract

**Background:**

Many medical family history (FH) tools are available for various settings. Although FH tools can be a powerful health screening tool in primary care (PC), they are currently underused.

**Objectives:**

This review explores the FH tools currently available for PC and evaluates their clinical performance.

**Methods:**

Five databases were systematically searched until May 2021. Identified tools were evaluated on the following criteria: time-to-complete, integration with electronic health record (EMR) systems, patient administration, risk-assessment ability, evidence-based management recommendations, analytical and clinical validity and clinical utility.

**Results:**

We identified 26 PC FH tools. Analytical and clinical validity was poorly reported and agreement between FH and gold standard was commonly inadequately reported and assessed. Sensitivity was acceptable; specificity was found in half of the reviewed tools to be poor. Most reviewed tools showed a capacity to successfully identify individuals with increased risk of disease (6.2–84.6% of high and/or moderate or increased risk individuals).

**Conclusion:**

Despite the potential of FH tools to improve risk stratification of patients in PC, clinical performance of current tools remains limited as well as their integration in EMR systems. Twenty-one FH tools are designed to be self-administered by patients.

KEY MESSAGES

- Currently available FH tools are heterogeneous in focus and performance.
- Analytical validity was poorly investigated. Sensitivity was found to be acceptable, while specificity was poor.
- Future FH tools should be self-administered by patients and integrated into EMR systems.

## Introduction

Taking a family history (FH) is the first step required to identify individuals at increased risk of various health conditions. A detailed FH assessment can identify entire families at risk. Early identification of individuals with increased risk allows health professionals to decrease their risk by following evidence-based guidelines in implementing medical interventions, lifestyle changes and increased disease surveillance [1,2].

Despite the potential merits of using FH tools, these are currently underused in clinical practice [3]. Several barriers have been reported that hamper the applicability of FH tools. The most significant barrier is the lack of time since clinicians typically have only 10 or less minutes per patient [4]. Other barriers include lack of proper training to collect and interpret FH, inaccurate information reported by patients and the FH tool itself not including standardised methodology [5–7]. Notably, most FH tools currently used in practice are not adequately validated [8–10]. These barriers can be overcome by using an FH tool that can collect FH in a structured way, organise data into a usable form, show good diagnostic performances, offer risk assessment (preferably based on an algorithm) and an evidence-based recommendation. The FH tool should also require an adequate completion time (patients report around 45 min to be the maximum acceptable time) [11].

Previous reviews of FH tools [1,4,7–10,12,13] concluded that FH tools can identify a relatively large proportion of people at increased risk that have not been identified before and are generally accurate [1,8,13]. Most FH tools currently used in practice are not validated against the standard reference (i.e. pedigree interview with a certified genetic counsellor) [8–10]. The implementation of FH tools into the public health system, however, requires a systematic evaluation of FH tools on the clinical validity and utility [7]. Importantly, currently, there are no specific guidelines to assess the usefulness of FH tools, however; several previous studies recommended to use the ACCE (analytical validity, clinical validity, clinical utility and ethical issues) framework [7,14,15] developed by the U.S Centre for Disease Control’s Office of Public Health Genomic (Centre for Disease Control Office of Public Health Genomics, OPGH, 2010).

The last systematic review suggested that an ideal FH tool would be self-administered by patients [9], integrated with EMR, easy to use, would comprise risk assessment based on incorporated algorithms and contain evidence-based management strategies. The authors concluded that FH tools evaluated in their study were not ready to be implemented [9]. We designed this new study to identify current FH tools for PC and to evaluate their clinical performance and characteristics relevant for use in the public health system.

## Methods

A study protocol was registered at the International Prospective Register of Systematic Reviews (PROSPERO) with a Registration number CRD42020134790 and is available from https://www.crd.york.ac.uk/prospero/display_record.php?ID=CRD42020134790. The systematic review follows the Preferred Reporting Items for Systematic Reviews and Meta-analysis (PRISMA) guidelines (Appendix 1, Supplementary Material).

### Search strategy and selection criteria

We systematically searched PubMed, EMBASE, Web of Science, and CINAHL from January 1970 to October 2020. The search was updated in May 2021, and no further articles were found. Keywords included: FH tool, family health history, genetic assessment, family genetic screening, FH or pedigree. For each key term, we found an appropriate MeSH term, which included 1) pedigree, 2) genetic testing and 3) FH taking. The full electronic search strategy is shown in Appendix 2 (Supplementary Material). Additionally, we hand-searched the included articles and previously reported reviews, and some additional sources (e.g. Google Scholar). Each full-text article was assessed for the inclusion/exclusion criteria (Table 1).

**Table 1.** Inclusion and exclusion criteria.

Inclusion criteria	Exclusion criteria
Original articles without any language restrictions Primary focussed on the development of the new FH tool An attempt is made to validate the FH tool on at least one aspect of the ACCE framework (validity and utility) The tool is applicable for either multifactorial (cardiovascular disease, diabetes and cancers) or one-factorial disorders (e.g. cystic fibrosis)	Systematic reviews, editorials, letters, opinions and unpublished studies The purpose of the FH tool is an only research use Articles describing a simple family history question (‘does this disease run in your family’?) Screening tool for detection of risk factors other than family history Paediatric and maternity FH tools

FH: family history; ACCE: analytical validity, clinical validity, clinical utility and ethical/legal/social implications

### Data extraction

We extracted the author’s name and year of the study, the FH tools’ name if available, the condition assessed, the time to complete (TTC), whether the tool was integrated with electronic health record (EMR) systems, whether it was patient administered, the number of questionnaire’s items and the setting in which the FH tool could be applied. The setting was extracted from the body of the study and not from the information on the recruitment setting. Additionally, we pooled the data on risk assessment (based on what model or guidelines the FH tools were based) and any recommendations offered within or after the FH's assessment. We have also extracted data on analytical and clinical validity, and clinical utility based on potential benefits and harms, according to the ACCE framework (Table 2) [15]. We included only data that reported on the psychological harm, not on ethical, legal and social issues. Stigmatisation, discrimination, risk to privacy and confidentiality were not included and are beyond this review. In some cases, the agreement’s kappa coefficient was not reported but we calculated it from the study’s information – if available.

**Table 2.** Description of the ACCE (analytical validity, clinical validity, clinical utility and ethical implications) framework^a^ elements, its meaning and data extraction.

Framework elements	Meaning	Data extraction
Analytical validity	An indicator of how accurately is the data reported (e.g. accurately reported illness in relatives, such as their relatedness, disease and age of onset)	Analytical sensitivity and specificity and described the comparator Other indicators that show that the data is accurate (e.g. repeated tests)
Clinical validity	Information how accurately the tool predicts disease risk	Clinical sensitivity and specificity and described the comparator Other indicators that show that the tool predicts the disease (agreement shown with kappa value)
Clinical utility	Potential benefits provided by using the FH tools	Identification of increased risk shown by the FH tools Behavioural or other improvements (e.g. new diet implementations and regular self-exam)
Ethical, legal and social implications	Issues with FH tool that might negatively affect patients, families and society	Psychological harm (e.g. scores on anxiety symptoms before and after the use of FH tool)

^a^Based on Yoon et al. [15] and adapted from Mishara and Weisstub [14] and Valdez et al. [7].

FH: family history.

### Analysis of the evaluation of the FH tools

Tools were evaluated on TTC, presence of integration with EMR systems, patient administration, risk-assessment ability, evidence-based management recommendations and ACCE framework (Table 2). Tools were considered to have an acceptable completion time if the mean TTC was less than 45 min (see Introduction). Values on sensitivity and specificity of 90% were considered to be good test characteristics, values of 80% were deemed acceptable and anything below that showed poor acceptability. Kappa on the agreement reports was supposed to be very good (kappa 0.81–1.00), good (kappa 0.61–0.80) and acceptable (kappa 0.41–0.60) [16]. Interclass correlation coefficient (ICC) was considered to show poor, moderate, good or excellent agreement if values were less than 0.5, between 0.5 and 0.75, between 0.75 and 0.9 and greater than 0.90, respectively [17].

## Results

**Figure 1.**
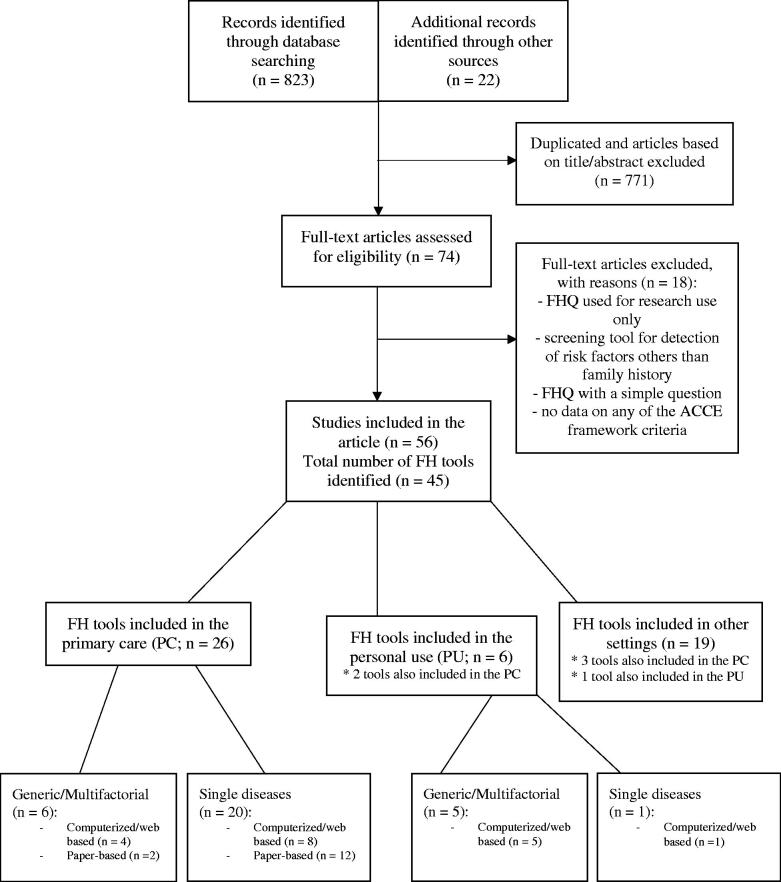
The selection process of included studies.

The search strategy yielded 823 results (Figure 1), and additionally, 22 articles were identified by going through the reference list of the included publications. After excluding the duplicates and articles not eligible for inclusion, 74 articles remained for a full-text read. Of these, 18 articles were excluded (Figure 1), and 56 studies reporting on 45 FH tools were included. Appendix 3 (Supplementary Material) describes all 45 of them, however in text and main tables, only PC tools are reviewed and evaluated.

### Main characteristics of the tools

Of the 45 reviewed FH tools [18–62], 26 were developed for primary care (PC). Of those, six were generic (i.e. identification of multiple diseases) and 20 were disease-specific (i.e. one disease or disease group). Furthermore, 12 were computerised, and 14 were paper-based. Reviewed tools varied in assessed conditions (from single disease to 98 assessed diseases) – computerised tools assessed more diseases than paper-based (Supplementary Table S1).

**Table 3.** Main results: items included.

Tool’s name	Personal health history	Health history of first-degree relative	Health history of second-degree relative	Health history of third-degree relative	Age relatives start of disease	Data on ethnicity	Health behaviour
**1. Generic/multifactorial**							
**1.1. Computerised/web based**							
Family Healthware^™^ [18]	X	X	X		X		X
VICKY [19]	X	X			X		
MeTree [20]	X	X	X	X	X		X
Walter's FHQ [21]		X	X	X	X	X	
**1.2. Paper-based**							
Qureshi's FHQ [22]	X	X	X		X	X	
Emery's FHQ [23]		X			X		
**2. Single disease**							
**2.1. Computerised/web based**							
RAGs [24]		X	X	X	X		
FHAT [25]		X	X	X	X		
Self-administered Q [26]		X	X		X	X	
GRACE [27]		X	X		X		
GRAIDS [28]	X	X	X	X	X	X	
CRA Health [29]	X	X	X	X	X		
Online Referral test [30]	X	X	X		X		
D&Q-based Web Interfaces [31]		X	X	X	X		
**2.2. Paper-based**							
Leggat’s FHQ [32]		X			X		
Houses’s FHQ [33]	X	X			X		
FCAT [34]		X	X	X	X		
PAT [35]		X	X	X	X	X	
A 21-Item FHQ [36]	X	X	X		X	X	
FH Form [37]		X	X		X		
FH-7 Q [38]		X	X	X	X		
RST [39]	X	X	X	X	X	X	
Pieper’s FHQ [40]		X			X		
Niendorf’s FHQ [41]		X	X	X	X		
A 4-Item Q [42]		X	X		X		
STELO [43]	X	X	X		X		

WICKY: Virtual Counsellor for Knowing Your Family History; FHQ: Family History Questionnaire; RAG: Risk Assessment in Genetics; FHAT: Family History Assessment Tool; GRACE: Genetic Risk in the Clinical Environment; GRAIDS: Genetic Risk Assessment in an Intranet and Decision Support; CRA health: cumulative risk assessment health. D&Q-based: Diagram and Questionnaire-based; FCAT: Familial Cancer Assessment Tool; PAT: Pedigree Assessment Tool; RST: Referral Screening Tool; STELO: Sindromi dei Tumori Ereditati Lynch e Ovaio/mammella.

### Items reported in the FH tools

FH tools differed in the reported sections/items; all tools assessed first-degree relatives (FDRs) (i.e. parents, siblings and children) and the relatives’ onset of the disease, 21/26 tools also assessed second-degree relatives (SDRs; i.e. grandparents, grandchildren, uncles, aunts, nephews, nieces and half-siblings), and 12/26 tools also assessed third-degree relatives (TDRs). Personal health history was reported in 11 FH tools and data on ethnicity in seven tools. Data on ethnicity was mostly reported for FH tools assessing cancer risk. Health behaviours (e.g. smoking and drinking) were assessed in two tools (Table 3).

**Table 4.** Main results: clinical value.

Tool’s name	Condition	Timing	Patient administration	EMR integration	Risk- assessment	Evidence-based recommendations	Evaluation on diagnostic performances
**1. Generic/multifactorial**							
**1.1. Computerised/web based**							
Family Healthware^™^ [18]	BB, OC, CC, CHD, diabetes and stroke	20 min	Yes	No	Yes	Yes	Inadequately assessed agreement (30–35 *vs.* 32–33%)^a^
WICKY [19]	various	15-30 min	Yes	No	No	No (PDF pedigree)	Acceptable analytical validity for FDR only (86% for FDR, 42% for SDR), inadequately assessed agreement (49 vs. 31%)^b^
MeTree [20]	98 diseases	20-27 min	Yes	Yes	Yes	Yes	NA
Walter's FHQ [21]	BC, CC, Diabetes, IHD	15-30 min	Yes	No	Yes	Yes	Good/acceptable sensitivity and specificity scores (81–98%)
1.2 Paper-based							
Qureshi’s FHQ [22]	various	NA	Yes	No	Yes	No	Acceptable and adequately assessed agreement in overall (*κ* = 0.52; 95% CI 0.40–0.64) and good agreement in premature CHD (90%; *κ* = 0.67; 95% CI 0.49–0.85)
Emery’s FHQ [23]	various	9 Items	Yes	No	Yes	Yes	Acceptable sensitivity (95%) and low specificity (54%)
**2. Single disease**							
**2.1. Computerised/web based**							
RAGs [24]	BC and OC	NA	No	No	Yes	Yes	Good and inadequately assessed agreement^c^ (median no. of correct referrals: 6 and pedigrees: 4)
FHAT [25]	BC and OC	NA	No	No	No	No	Good sensitivity (94%) and poor specificity (51%)
Self-Administered Q [26]	BC and OC	NA	Yes	No	Yes	Yes	Adequately assessed low agreement (IC*C* = 0.63)
GRACE [27]	BC	NA	Yes	No	Yes	Yes	NA
GRAIDS [28]	BC, OC, CC and EC	28 min	No	No	Yes	Yes	NA
CRA Health [29]	hBC and hOC	NA	Yes	No	Yes	Yes	NA
Online Referral test [30]	Lynch syndrome	NA	Yes	No	Yes	Yes	Good sensitivity for affected Lynch syndrome carriers (91%), poor for affected and non-affected carriers (73%)
D&Q-based Web Interfaces [31]	CC	*D* = 81 s *Q* = 81 s	Yes	No	Yes	Yes	NA
**2.2. Paper-based**							
Leggat’s FHQ [32]	BC and CC	NA	Yes	No	Yes	Yes	Acceptable and adequately assessed agreement for BC (ICC = 0.58), but not for colon (ICC = 0.80)
Houses’s FHQ [33]	CC	NA	Yes	No	Yes	Yes	Good/acceptable and inadequately assessed agreement (5% discrepancy from the CC prevalence)^d^
FCAT [34]	BC	10 min	Yes	No	Yes	Yes	Good sensitivity (93%), acceptable specificity (83%)
PAT [35]	hBC	NA	No	No	Yes	Yes	Good sensitivity (100%), specificity (93%)
A 21-Item FHQ [36]	Cardiovascular	15 min	Yes	No	Yes	No	NA
FH Form [37]	BC, OC and CC	NA	Yes	No	Yes	No	NA
FH-7 Q [38]	BC	7 items	Yes	No	Yes	Yes	Acceptable and adequately assessed agreement (ICC = 0.84), acceptable sensitivity (87.6%) and poor specificity (56.4%)
RST [39]	hBC and hOC	NA	Yes	No	Yes	Yes	Good test-retest reliability (*k* = 0.75), acceptable sensitivity (81.2%) and good specificity (91.9%)
Pieper’s FHQ [40]	CC	3 items	Yes	No	Yes	No	Inadequately assessed analytical validity^e^
Niendorf’s FHQ [41]	Hereditary cancers	7 items	Yes	No	Yes	Yes	Acceptable and adequately assessed agreement (IC*C* = 0.87)
A 4-Item Q [42]	CC	4 items	No	No	NA	NA	Good agreement (*k* = 0.82), but inadequately assessed^f^ analytical validity for first item only (others could not be assessed)
STELO [43]	Inherited cancer syndromes	9 items	Yes	No	Yes	Yes	Acceptable and inadequately assessed^g^ agreement (ICC = 0.77) and acceptable sensitivity (88.5%) and poor specificity (52.3%)

NA: not assessed; BC: breast cancer; hBC: hereditary breast cancer; CC: colorectal cancer; OC: ovarian cancer; EC: endometrial cancer; CHD: coronary heart disease; FDR: first-degree relative; ICC: interclass correlation coefficient; Κ: kappa value; WICKY: Virtual Counsellor for Knowing Your Family History; FHQ: Family History Questionnaire; RAG: Risk Assessment in Genetics; FHAT: Family History Assessment Tool; GRACE: Genetic Risk in the Clinical Environment; GRAIDS: Genetic Risk Assessment in an Intranet and Decision Support; CRA health: cumulative risk assessment health. D&Q-based: Diagram and Questionnaire-based; FCAT: Familial Cancer Assessment Tool; PAT: Pedigree Assessment Tool; RST: Referral Screening Tool; STELO: Sindromi dei Tumori Ereditati Lynch e Ovaio/mammilla; MFHP: My Family Health Portrait.

Notes. ^a^Compared with risk-stratifaction. ^b^Compared with MFHP. ^c^Hypothetical cases. ^d^compared with NCRAS (172 *vs.* 260 per 100,000 patients). ^e^Evaluation of an uptake of the questionnaire as a comparator. ^f^general practitioners' responses. ^g^Compared to the clinical records.

### Evaluating FH tools based on TTC, patient administration, EMR systems integration, risk-assessment ability, presence of evidence-based recommendations, analytical and clinical validity and clinical utility

#### TTC, patient administration and EMR system integration

Information on TTC was reported in eight reviewed tools, while six tools reported only the number of items (range: 3–9 items), which gave at least some information about the length of the questionnaire. In all reviewed tools, timing was found satisfactory (mean = 17.9 min; range 81 s − 28 min). All six generic/multifactorial were patient-use [18–21,23,63], and out of 20 single disease tools, 15 were patient-use and 5 were administered by physician [24,25,28,35,42]. Only one tool (MeTree) enables the FH report to be included in the patient’s EMR [20,64] (Table 4).

**Table 5.** Recommended tools for primary care and personal use.

FH tool	Time to complete	Patient administration	EMR integration	Risk- assessment	Recommendations	Validity
**1. Generic/multifactorial**						
**1.1. Computerised/web based**						
Walter’s FHQ [21]	X	X	–	X	X	X
**1.2. Paper-based**						
Emery’s FHQ [23]	X	X	–	X	X	X**
**2. Single diseases**						
**2.1. Paper-based**						
FCAT [34]	X	X	–	X	X	X
PAT [35]	–*	–	–	X	X	X
FH-7Q [38]	X***	X	–	X	X	X**
RST [39]	–*	X	–	X	X	X
Niendorf’s FHQ [41]	X***	X	–	X	X	X
STELO [43]	X***	X	–	X	X	X**

*Not assessed. **: acceptable sensitivity, low specificity. ***: only the number of item reported.

FH: family history; FHQ: Family History Questionnaire; FCAT: Familial Cancer Assessment Tool; PAT: Pedigree Assessment Tool; RST: Referral Screening Tool; STELO: Sindromi dei Tumori Ereditati Lynch e Ovaio/mammilla; EMR: Electronic Medical Records.

### Risk assessment ability and presence of evidence-based management recommendations

Risk assessment was included in 23 tools, and evidence-based recommendations were offered in 19 tools. Risk assessment and evidence-based recommendations in PC were included in five tools. In WICKY^™^, risk assessment or recommendations are not offered; however, a PDF pedigree can be printed and offered to the clinician instead [19] (Supplementary Table S1 and Table 4 ).

#### Analytical and clinical validity

Studies used different comparators for analytical validity: repeated responses, relative’s self-reported disease status, general practitioners’ (GPs) notes, and other validated FH tools. The gold standard for clinical validity was, in most cases, a pedigree interview obtained by a genetic counsellor; however, in some cases, other comparators (surgical oncologists, GPs, trained clinical nurses, clinical records and studies with comparable risk-stratifications) were employed (Supplementary Table S1 and Table 4).

Analytical validity was reported for three tools [19,40,42]. It showed to be inadequately assessed in Pieper’s FHQ [40] and in a 4-item Q [42]; while in WICKY^™^, values showed acceptable analytical validity for FDR only [19] (Supplementary Table S1). Clinical validity was reported for 17 tools. Good/acceptable sensitivity values were found for nine tools [21,23,25,30,34,35,38,39,43]. The online referral test showed good sensitivity for affected Lynch syndrome carriers and low for affected and non-affected carriers [30]. Specificity was reported to be good/acceptable in four tools [21,34,35,39] and low in four tools [23,25,38,43]. Agreement between FH and gold standard was commonly inadequately reported and assessed. It was reported in 11 tools while it was found to be adequately assessed in only five of them and acceptable in four [22,26,32,38].

### Clinical utility (benefits and adverse effects)

Reports on clinical utility addressed the benefits of identifying patients with an increased risk, psychological impact, behavioural change and adverse effects (Supplementary Table S1). For reviewed tools, benefits were reported for 14 FH tools, of which the majority have benefits in terms of identifying patients with increased risk. Thus, reviewed tools identified 6.2–84.6% of high and/or moderate or increased risk individuals [18,20–22,26,32,33,35–37,40,41] and 3.6% of individuals eligible for genetic testing [29]. Benefits included increased reassurance, certainty about their familial risk and/or certainty about referral [30] and raised awareness of disease risk [20]. Improvements were also observed within the family practice (e.g. improved understanding and easier practice) [20].

Studies suggest that FH collection does not lead to psychological distress [22,27,33]. Only one study reported a possible risk [22]; specifically, the study reported that due to the FH screening questionnaire, patients had higher anxiety symptoms but only for the first and second week after the intervention (*F* = 6.4; df = 1.73; *p* = .014); short-term psychological distress did not persist after a three-month follow-up.

## Discussion

### Main findings

This article explores and summarises the main characteristics of the FH tools and evaluates their simplicity of use, clinical performance and potential for integration with EMR systems. Currently available FH tools can be used in PC, at home and in other settings (clinical genetic counselling, cancer management and internal medicine (Appendix 3, Supplementary Material)). Tools in PC are, in most cases, oriented towards a single disease (20/26), estimate familial risks for cancer (18/23) and are usually in paper format (13/23). In general, the time needed to complete the FH tool, which is related to the simplicity of use, was poorly reported. However, most FH tools can be completed in less than 30 min.

As FH tools share the characteristic of identifying genetic predisposition with genetic tests, the ACCE framework might be the best current option to evaluate scientific data reported by FH tools. Our evaluation showed that advanced tools are not adequately validated. Only a few assessed analytical validity, and in only one was validation assessed adequately with the ‘optimal’ gold standard (i.e. pedigree interview with a certified genetic counsellor) [19]. In general, clinical validity was reported more often; however, tools were commonly inadequately reported and assessed. When correctly assessed, sensitivity was acceptable in most cases, while the specificity was found in half of them to be poor. Therefore, patients might receive potentially unnecessary consultations with a genetic counsellor. Most tools showed the capacity to successfully identify individuals with increased risk and taking FH does not pose psychological harm to those patients identified with increased risk.

### Comparison with existing literature

Our results are in line with findings from Reid et al. that currently available FH tools present potential benefits in terms of their capacity to successfully identify individuals with an increased risk and increase individual’s risk perception about their familial risk (Supplementary Table S1) [8]. Although learning about an increased risk might initially trigger stress in patients, short-term psychological distress in the reported studies did not persist during the follow-up of a few months [22]. Offering psychological interventions while ensuring that the patient is accurately referred would appear sensible. Notably, a higher level of knowledge regarding the identified condition was associated with less fear [40]. Patients that learnt about the familial disease for the first time increased their personal risk perception [20,30,49], were more aware of the possible prevention strategies and visited their doctors less frequently [40,53]. Furthermore, developed FH tools do not have reports on ethical and legal implications, including privacy, confidentiality, ownership of data and informed consent.

Although computerised/web-based FH tools present many benefits (cost less, are completed faster, the data is instantly digitalised), analyses have shown that in the PC setting, electronic tools provide little benefit over traditional paper-based assessment [2], and can also result in a lower response rate [65]. Taking detailed FH is indeed a time-consuming process, and most clinicians only have a few minutes to ask questions about a patients’ FH of the disease. However, this usually occurs once, at the patient’s first visit [3], decreasing the number of necessary updates and gathering accurate information [13].

On the other hand, a recent study has shown that patients are comfortable sharing their family health information with their physicians over the internet [66]. As reported previously [4,7], electronic web-based FH tools are a promising approach since those tools can integrate with other clinical and office systems and make physicians better for accurate referral decisions.

Previous studies [9,13] have observed that it was challenging to compare available tools due to format heterogeneity, varying approaches, the setting of the tool and the number of diseases assessed. They concluded that it is impossible to recommend any of the identified tools. Unfortunately, even if done eight years later, our review reports similar findings. From 2014, nine more tools were developed or additionally explored in other studies [19,20,31,41–43,45–47]. One tool (MeTree) is now available for online transfer to EMR; however, it was not validated [9,20]. Currently, there still is no FH tool that would report and adequately assess analytical and clinical validity, offer algorithm-based risk assessment, evidence-based recommendations and adequate TTC. Based on the evaluation of currently available FH tools, we list those which provide the best evidence for implementation in PC (Table 5).

Our list of suggested FH tools differs from the list provided in the review of de Hoog et al. [9]. Only FH tool My Family Health Portrait was indicated in both reviews, however, this tool is recommended only for personal use and not for PC assessment. As emphasised in a review from Ginsburg in 2019 [1], we agree that FH tools should be patient-completed, preferably electronic-web based and comparable with EMRs. Moreover, it should be completed in less than 30 min, including at least FH information on first and SDRs and their ages, personal information (age, gender and ethnicity) and risk assessment based on clinical significance and point of care recommendations. We recommend future FH tools to report on analytical and clinical validity and assess clinical utility (benefits and risks).

#### Methodological considerations

Interpretation of the results in this review should be considered in light of some study limitations. The lack of adequate assessment of the analytical and clinical validity is perhaps this study’s most significant and troubling result. For more accurate results, data from patients’ medical records and their reported relatives would need to be obtained. Thus, most of the data comes from the patients’ reports on the relative’s illnesses, which lowers the reports’ accuracy since certain diseases, such as breast cancer are more accurately reported than others (e.g. uterine cancer [67]). Therefore, we suggest that FH tools should be evaluated on their capacity to identify certain high-risk groups. An additional limitation of this review was screening for relevant articles. Regardless of the rigorous systematic approach in exploring the articles, after additional screening for relevant articles in the previously reported reviews and further screening in other electronic sources, we found an additional 22 articles. This shows that our initial search strategy was not accurately set. Some articles are titled ‘risk-assessment’ rather than ‘family history tool/pedigree,’ which was our primary search key term. However, after the additional screening, we believe that we have covered the majority of the currently utilised FH tools if not all.

### Implications for research and practice

Despite many FH tools currently available, their routine clinical or personal use is not yet advised, mainly because of the lack of proper validation. Some of the critical issues should be addressed. The agreement between FH tool and the gold standard (clinical interview with the genetics) should be assessed and reported. The Secretary’s Advisory Committee on Genetic Testing (SACGT) [68] recommended assessing benefits and risks, and this is what we have done with the ACCE framework. Albeit developed FH tools do not have reports on ethical and legal implications on the last ACCE framework criteria, we were able to evaluate them on analytical and clinical validity, and assess potential benefits and harms. Though this is not the perfect instrument to evaluate FH tools; it is currently the best one we have. In our literature review, only a few studies have assessed the uptake rates for genetic counselling. We recommend future studies be attentive to how many of the ‘increased-risk patients’ pursue genetic counselling after the referral.

## Conclusion

Despite the potential of FH tools to improve risk stratification of patients in PC, evidence on the clinical performance of current tools remains limited as well as their integration in EMR systems. Twenty-one FH tools are designed to be self-administered by patients.

## Supplementary Material

Table S1

Supplemental Appendix 1

Supplemental Appendix 3

Supplemental Appendix 2
