# Supplementary material for: Family history tools for primary care: A systematic review
Source: Eur J Gen Pract. 2022 May 5;28(1):75–86. doi: 10.1080/13814788.2022.2061457 (PMC9090347; doi:10.1080/13814788.2022.2061457)
Supplement: Table S1 [file IGEN_A_2061457_SM3923.docx]

Table S1. Main results: Overview of the FH tools for Primary Care

| Reference or tool name | Condition | Main tools characteristics | Risk assessment? | Recommendations? | Validity | Clinical utility |
| --- | --- | --- | --- | --- | --- | --- |
| 1. GENERIC/MULTIFACTORIAL | | | | | | |
| - 1. COMPUTERIZED/ WEB BASED | | | | | | |
| Yoon et al., 2009, Rubinsten et al. 2011; Ruffin et al. 2011; O’Neill et al. 2009 (Family Healthware™) (18) | Colon, breast and ovarian cancer, CHD, diabetes and stroke | a) pedigree-oriented  b) patient-completed | Based on the predefined framework (low, moderate, high) | Based on various factors (level of risk, sex, age, health behaviours, e.g., diet), lifestyle changes and screening | *Clinical validity* (comparator*:* studies with comparable risk-stratification); good agreement (CHD: 30%-35% vs 32%-33%; diabetes: 8%-12% vs 7%-16%) | *Identification of an increased risk:* - 34% moderate/high risk for cancer; *Behaviour change:* 3% eating healthier and 4% increase in PA after personalized risk-tailored vs age- and sex- specific messages |
| Wang et al. 2015; Diez et al. 2019 (VICKY) (19) | Various cancers, heart disease, diabetes, hypertension, stroke | a) pedigree-oriented  b) patient-completed | No | Patient receives a pedigree chart (PDF), which can be used for healthcare  providers | *Analytical validity (comparator: pedigree with GS)*  - VICKY identified 86% of FDR, and 42% of SDR; (accuracy for both: 55%)  *Clinical validity* (comparator: MFHP): VICKY identified a greater number of health conditions overall (49% VICKY vs 31% MFHP, p=.008); hypertension (47% vs 15%, p=.001) and type 2 diabetes (54% vs 22%; p=.004) | NA |
| Orlando, 2013; Wu, 2013; 2016; Buchanan et al. 2015; Goldstein et al. 2019 (MeTree) (20) | 48 diseases | a) pedigree-oriented  b) patient-completed  c) EMR | Based on guidelines for specific disease (avarage risk and several risk levels) | Based on the disease and the risk (GS, testing, no recommendation) | NA | *Identification of an Increased risk:* higher identification than risk management (1.1% before and 16.1% after MeTree for increased-risk group; for average-risk 99.2% before and 99.5% after MeTree; 10% met criteria for an increased risk before, and after MeTree, 80.7%)  *Behavioural change:* 85% raised awareness of disease risk  - 86% changed how they think about their health; Other impact: - among the providers, 86% improved their practice, 64% improved their understanding of FHH, and 79% made practice easier |
| Walter et al. 2013 (21) | Diabetes, ischaemic heart disease, breast cancer, colorectal cancer | a) pedigree-based  b) patient-completed | Based on the evidence appraisals or  guidelines (increased risk) | A nurse visits  for diagnostic tests  or a GP  visit for referral  screening tests | *Clinical validity* (comparator: pedigree by a trained clinical nurse): Diabetes: sensitivity = 98%, specificity = 94%; IHD: sensitivity = 93%, specificity = 81%; BC: sensitivity = 81%, specificity = 83%; CC: sensitivity = 96%, specificity = 88%, AUC – ROC Curve of 0.90 for males and 0.89 for females | *Identification of an Increased risk: -* 32% were at increased risk of one or more marker conditions (diabetes 18.9%,  ischaemic heart disease 13.3%, breast cancer  6.2%, colorectal cancer 2.2%). |
| - 1. PAPER-BASED | | | | | | |
| Qureshi et al. 2001; 2005; 2012 (FHQ) (22) | Multifactorial | a) pedigree-oriented  b) patient-completed | Based on the current disease guideliness; higher than population risk | No | *Clinical validity* (comparator: genetic interview by trained researchers): - 77% agrmt overall (κ=0.52; 95% CI 0.40–0.64); 90% agrmt in the premature CHD (90%; κ=0.67; 95% CI 0.49 to 0.85) | *Identification of an increased risk:* - high risk (64.1%) and moderate risk for at least one CHD disease (84.6%); - more identification at high-risk vs patient records (M 4.8% vs 0.3%); *Psychological impact:* - anxiety scores with vs without FH tool were similar |
| Emery et al. 2014; Houwink et al. 2019 (23) | Multifactorial (cancers, IHD, type 2 diabetes) | a) disease-oriented  b) patient-completed | Based on guidliness on specific disease (positive, negative) | A more detailed  assessment of their FH if positive | *Clinical validity* (comparator: pedigree by GS)  - AUC (84.6%); sensitivity (95%); specificity (54%) to identify participants with increased risk of any condition; gender specificity (men 63%; women 49%) |  |
| 1. SINGLE DISEASES | | | | | | |
| - 1. COMPUTERIZED/ WEB BASED | | | | | | |
| Emery et al. 1999; 2000 (RAGs) (24) | Breast and ovarian cancer | a) pedigree-oriented  b) clinician-completed | Based on Claus model, data from case-control study of 4730 BC cases (low, moderate, high) | Low risk managed in PC, moderate at breast unit, high risk at genetics clinic | *Clinical validity* (comparator: pedigrees drawn with a) Cyrillic, established pedigree program, and b) pen and paper): - median no. of correct referrals: 6 and pedigrees: 4 | NA |
| Gilpin et al. 2000 (FHAT) (25) | Breast and ovarian cancer | a) pedigree-oriented  b) clinician-completed | Not yet available | No | Clinical validity (comparator: testing for BRCA1 and BRCA2): sensitivity 0.94, specificity 0.51, PP 0.31, NP 0.97 | NA |
| Hughes et al. 2003 (A self-administered questionnaire) (26) | Breast and ovarian cancer | a) pedigree-oriented  b) patient-completed | Based on study criteria (No FH, insignificant FH, significant potentially high-risk) | Mammography, breast examinations, genetic counselling, genetic testing | *Clinical validity (*comparator: pedigree by surgical oncologist):  - 62.7% accuracy | *Identification of an increased risk:* - 9.4% had a significant family history (of those 62.7% were confirmed by the pedigrees) |
| Braithwaite et al. 2005 (GRACE) (27) | Breast cancer | a) pedigree-oriented  b) patient-completed | Based on Claus model using regional  Guidelines (low, moderate, high) | Breast awareness, use of mammography and genetic testing | NA | *Psychological impact:* - difference in risk perception and cancer related worries (between GRACE and nurse counselling: not statistically significant (P>0.05) for both outcomes |
| Emery et al. 2005; 2007 (GRAIDS) (28) | Breast, ovarian, colorectal, and endometrial cancers | a) pedigree-oriented  b) clinician-completed | Based on Claus model and Guidelines from the Regional Genetic Centre (average, moderate and high) | Further assessment and advice on screening for moderate and high risk;for high advice for genetic testing | NA | *Identification of an increased risk:* - more referrals than practise (MD= 3.2; CI 1.2–4.8; p=0.001); referrals more consistent with guidelines (OR 5.2; CI 1.7–15.8, p=0.006).  *Psychological impact:* - lower CWS scores after I (MD= 1.4; 95% CI 2.64 to 0.23, p=0.02); no differences in knowledge  *Other impact:* - the intervention increased GPs’ confidence in managing familial cancer |
| Ozanne et al. 2009; Williams 2012 (CRA Health ex. HughesRiskApp) (29) | Hereditary breast and ovarian cancer | a) pedigree-oriented  b) patient-completed | Based on BRCAPRO, Myriad (Ozanne), EPA Framework (Williams) | Advice on attending GS and clinical decision support | NA | *Identification of an increased risk:*  *-* the tool has successfully identified 3.6% individuals eligible for genetic testing |
| Dekker et al. 2013 (Online referral test) (30) | Lynch syndrome | a) disease-oriented  b) patient- and clinician- completed | Based on the Dutch guidelines on hereditary  CRC; low, moderate and high | High risk - GS; moderate risk - surveillance colonoscopies | *Clinical validity* (comparator: pedigree)  - sensitivity was 91% for mutation carriers with CRC (n = 164) and 73% for all affected and non-affected mutation carriers (n = 420) | *Psychological impact:* 71% of 256 users reported that the referral test increased reassurance, certainty about their familial risk and/or certainty about referral. |
| Schultz et al. 2015 (Diagram and questionnaire-based web interfaces) (31) | Colorectal cancer | a) pedigree-oriented  b) patient-completed | Based on the New Zealand guidelines;  average, slightly increased, moderately increased and potentially high lifetime risk | Screening and surveillance of  patients with an increased risk of CRC | NA | NA |
| - 1. PAPER-BASED | | | | | | |
| Leggatt et al. 1993; Wallace et al. 2014 (FHQ) (32) | Breast and colorectal cancer | a) disease-oriented  b) patient-completed | Made by GPs following cancer genetic criteria (low, moderate, high) | Screening for those with an increased risk | *Clinical validity (comparator:* interview/telephone call with GS) - agreement for BC was 58.3%*;* for colon cancer was 80% | *Identification of an increased risk:* - 17% with increased risk |
| House et al. 1999; Rose et al. 2004 (FHQ) (33) | Colorectal cancer | a) disease-oriented  b) patient-completed | High risk using Amsterdam criteria; moderate and low risk using local guidelines | Screening for colonoscopy for high risk | *Clinical validity* (comparator: CC prevalence): *-* only 5% discrepancy in prevalence compared with NCRAS (172 vs 260 per 100.000 patients)  (comparator: GS review): - 5 inter-mediate risk patients were assigned to high risk group | *Identification of an increased risk:* - de novo identified in the high (20%) and moderate (39%) group; *Psychological impact:* - no difference observed in STAI and IES |
| Benjamin et al. 2003 (FCAT) (34) | Breast cancer | a) disease-oriented  b) patient-completed | Based on a broad agreement (low>16%, medium 16-25%, high<25%) | If positive, referral to a FH clinics or genetic department (more than 25%) | *Clinical validity* (comparator: interview with GS): - sensitivity was 92% (95% Cl, 84-97%), specificity was 83% (95% Cl, 54-94%); PPV 0.97 (95% Cl, 90-99%); NPV 0.68 (95% Cl, 45-68%) | NA |
| Hoskins et al. 2006; 2010 (PAT) (35) | Hereditary breast cancer | a) pedigree-oriented  b) clinician-completed | Based on the study’s scoring (potentially increased risk, high and low BRCA risk) | GS and referral for BRCA gene mutation analysis | *Clinical validity* (comprator: Frank model): Sensitivity 100%, specificity 93%, PPV 63%, NPV 100% | *Identification of an increased risk*: -18.9% of potentially increased risk; 2.2% of a high probability that BRCA mutation is present; The PAT did better than Gail model in identifying those at high risk) |
| MacLeod et al. 2007 (A 21-item FHQ) (36) | Inherited cardiovascular disease | a) pedigree-oriented  b) patient-completed | Based on the Scheuner et. al 1997 model (high, moderate, average) | No | NA | *Identification of an increased risk:* 64.1% of patients at high risk, 84% at moderate risk; 23% of questionnaires showed the same information as GPs notes |
| Murff et al. 2007 (FH form) (37) | Breast, ovarian and colorectal cancer | a) pedigree-oriented  b) patient-completed | Breast and ovarian based on the literature and CC based on the guidelines (increased risk) | No | NA | *Identification of an increased risk -* % subjects were screen as high risk; - more at-risk individuals identified: 29 vs 19 in the chart; more information on age as compared to the chart (81% vs 40% in the medical chart) |
| Ashton-Prolla et al. 2009 (FH-7 questionnaire) (38) | Breast cancer | a) disease-oriented  b) patient-completed | Based on ASCO criteria for increased risk | Positively answered to one question, referred for further assessment | Clinical validity (comparator: pedigree)  - sensitivity (87.6%) and specificity (56.4%)  - ICC for at least one positive answer was 0.84 | NA |
| Bellcross et al. 2009 (RST) (39) | Hereditary risk for breast/ovarian cancer | a) pedigree-oriented  b) patient-completed | Based on US Preventive Service Task Force (2005); positive high risk; negative, low risk | Referral for GS and testing for BRCA1/2 mutations | *Clinical validity (comparator:* pedigree by GS)  - sensitivity = 81.2%; specificity = 91.9%; area under the curve = 0.87%  - Test-retest reliability: concordance of 156/2464 was 96% (k = 0.75) | NA |
| Pieper et al. 2012 (40) | Colorectal cancer | a) pedigree-oriented  b) patient-completed | Based on the Network  against colorectal cancer guiideliness (one positive answer: increased risk) | NA | *Analytical validity (comparator:* an evaluation of an uptake of the questionnaire): - more positive responses on question 1 at t1 than at t3 (13% vs. 11%); - more “Don’t know” answers at t1 (t1: 19% vs. t2: 4%) in question 2 | *Identification of an increased risk:* - the tool identified 16% patients with increased risk; *Other impact:* - patients identified at increased risk for the first time had significantly less frequent physician contact (GP: 20% vs. 34%, gastroenterologist: 8% vs. 21%) |
| Niendorf et al. 2016 (41) | Hereditary cancer syndrome | a) disease-oriented  b) patient-completed  c) + telephone interview | Based on the Hampel Criteria; high, moderate and average risk | High risk received advice for GC, GS, list of local G services, and current guidelines | *Clinical validity* (comparator: GC):  - agrmt for an increased risk was 87% (n=500) | Identification: - 66.4% were found to be at high risk, 20.4% at moderate and 13.2% at average risk for hereditary cancer syndrome |
| Koné et al. 2018 (A 4-item questionnaire) (42) | Colorectal cancer | a) pedigree-oriented  b) clinician-completed | No | No | *Analytical validity (*comparator: general practitioners' responses): *-* good agrmt on Q1 (first-relative diagnosed with CC): kappa 0.82; Cl 0.58-1.10)*;* - Q 2-4 could not be validated with this methodology | NA |
| Mariani et al. 2020 (STELO) (43) | Inherited cancer  Syndromes | a) pedigree-oriented  b) patient-completed | Scientific literature and disease guidelines; increased risk | One positive answer, send to the GS | *Clinical validity (comparator: clinical records):*  - 76.5% agrmt; sensitivity 88.5%, specificity 52.3% | NA |

Abbreviations. NA – not assessed. FH – family history. OR – odds ratio. GP – general practitioner. MD – mean difference. CWS – cancer worry scale. GS – genetic screening. GC – genetic counselor. BC – breast cancer. CC – colorectal cancer. CHD – coronary heart disease. CRC – IHD - ischaemic heart disease. MFHP - My Family Health Portrait. PA – physical activity. agrmt – agreement. PPV – positive predictive valua. NPV – negative predictive value. RST – referral screening tool. WICKY – Virtual Counsellor for Knowing Your Family History. RAG – Risk Assessment in Genetics. FHAT – Family History Assessment Tool. GRACE – Genetic Risk in the Clinical Environment. GRAIDS – Genetic Risk Assessment in an Intranet and Decision Support. CRA health – cumulative risk assessment health. FCAT – Familial Cancer Assessment Tool. PAT – Pedigree Assessment Tool. RST – Referral Screening Tool. STELO – Sindromi dei Tumori Ereditati Lynch e Ovaio/mammella. K – kappa. ROC curve – receiver operating characteristics. ICC – interclass correlation coefficient.
