# Supplementary material for: Family history tools for primary care: A systematic review
Source: Eur J Gen Pract. 2022 May 5;28(1):75–86. doi: 10.1080/13814788.2022.2061457 (PMC9090347; doi:10.1080/13814788.2022.2061457)
Supplement: Supplemental Appendix 3 [file IGEN_A_2061457_SM7905.docx]

Appendix 3. Studies reporting FH tools sorted by the year of the publishment

| # | Reference or tool name | Condition | Main tools characteristics | Risk assessment? | Recommendations? | Validity | Clinical utility |
| --- | --- | --- | --- | --- | --- | --- | --- |
| 1. PRIMARY CARE TOOLS | | | | | | | |
| 1.1. GENERIC/MULTIFACTORIAL | | | | | | | |
| 1.1.1. COMPUTERIZED/ WEB BASED | | | | | | | |
| 1 | Yoon et al., 2009, Rubinsten et al. 2011; Ruffin et al. 2011; O’Neill et al. 2009 (Family Healthware™) (16) | Colon, breast and ovarian cancer, CHD, diabetes and stroke | a) pedigree-oriented  b) patient-completed | Based on the predefined framework (low, moderate, high) | Based on various factors (level of risk, sex, age, health behaviours, e.g., diet), lifestyle changes and screening | *Clinical validity* (comparator*:* studies with comparable risk-stratification); good agreement (CHD: 30%-35% vs 32%-33%; diabetes: 8%-12% vs 7%-16%) | *Identification of an increased risk:* - 34% moderate/high risk for cancer; *Behaviour change:* 3% eating healthier and 4% increase in PA after personalized risk-tailored vs age- and sex- specific messages |
| 2 | Wang et al. 2015; Diez et al. 2019 (VICKY) (17) | Various cancers, heart disease, diabetes, hypertension, stroke | a) pedigree-oriented  b) patient-completed | No | Patient receives a pedigree chart (PDF), which can be used for healthcare  providers | *Analytical validity (comparator: MFHP)*  - VICKY identified 86% of FDR, and 42% of SDR; (accuracy for both: 55%)  *Clinical validity* (comparator: pedigree with GS and compared to MFHP): VICKY identified a greater number of health conditions overall (49% VICKY vs 31% MFHP, p=.008); hypertension (47% vs 15%, p=.001) and type 2 diabetes (54% vs 22%; p=.004) | NA |
| 3 | Orlando, 2013; 2020; Wu, 2013; 2016; Buchanan et al. 2015; Goldstein et al. 2019 (MeTree) (18) | 98 diseases | a) pedigree-oriented  b) patient-completed  c) EMR | Based on guidelines for specific disease (avarage risk and several risk levels) | Based on the disease and the risk (GS, testing, no recommendation) | NA | *Identification of an Increased risk:* higher identification than risk management (1.1% before and 16.1% after MeTree for increased-risk group; for average-risk 99.2% before and 99.5% after MeTree; 10% met criteria for an increased risk before, and after MeTree, 80.7%)  *Behavioural change:* 85% raised awareness of disease risk  - 86% changed how they think about their health; Other impact: - among the providers, 86% improved their practice, 64% improved their understanding of FHH, and 79% made practice easier |
| 4 | Walter et al. 2013 (19) | Diabetes, ischaemic heart disease, BC, CC | a) pedigree-based  b) patient-completed | Based on the evidence appraisals or  guidelines (increased risk) | A nurse visits  for diagnostic tests  or a GP  visit for referral  screening tests | *Clinical validity* (comparator: pedigree by a trained clinical nurse): Diabetes: sensitivity = 98%, specificity = 94%; IHD: sensitivity = 93%, specificity = 81%; BC: sensitivity = 81%, specificity = 83%; CC, sensitivity = 96%, specificity = 88%, AUC – ROC Curve of 0.90 for males and 0.89 for females | *Identification of an Increased risk: -* 32% were at increased risk of one or more marker conditions (diabetes 18.9%,  ischaemic heart disease 13.3%, breast cancer  6.2%, colorectal cancer 2.2%). |
| 1.1.2. PAPER-BASED | | | | | | | |
| 5 | Qureshi et al. 2001; 2005; 2012 (FHQ) (20) | Multifactorial | a) pedigree-oriented  b) patient-completed | Based on the current disease guideliness; higher than population risk | No | *Clinical validity* (comparator: genetic interview by trained researchers): - 77% agrmt overall (κ=0.52; 95% CI 0.40–0.64); 90% agrmt in the premature CHD (90%; κ=0.67; 95% CI 0.49 to 0.85) | *Identification of an increased risk:* - high risk (64.1%) and moderate risk for at least one CHD disease (84.6%); - more identification at high-risk vs patient records (M 4.8% vs 0.3%); *Psychological impact:* - anxiety scores with vs without FH tool were similar |
| 6 | Emery et al. 2014; Houwink et al. 2019 (21) | Multifactorial (cancers, IHD, type 2 diabetes) | a) disease-oriented  b) patient-completed | Based on guidliness on specific disease (positive, negative) | A more detailed  assessment of their FH if positive | *Clinical validity* (comparator: pedigree by GS)  - AUC (84.6%); sensitivity (95%); specificity (54%) to identify participants with increased risk of any condition; gender specificity (men 63%; women 49%) |  |
| 1.2. SINGLE DISEASES | | | | | | | |
| 1.2.1. COMPUTERIZED/ WEB BASED | | | | | | | |
| 7 | Emery et al. 1999; 2000 (RAGs) (22) | Breast and ovarian cancer | a) pedigree-oriented  b) clinician-completed | Based on Claus model, data from case-control study of 4730 BC cases (low, moderate, high) | Low risk managed in PC, moderate at breast unit, high risk at genetics clinic | *Clinical validity* (comparator: pedigrees drawn with a) Cyrillic, established pedigree program, and b) pen and paper): - median no. corrected pedigrees: RAGS (6/6), Cyrillic (3/6), and pen-paper (3/6)) | NA |
| 8 | Gilpin et al. 2000 (FHAT) (23) | Breast and ovarian cancer | a) pedigree-oriented  b) clinician-completed | Not yet available | No | Clinical validity (comparator: testing for BRCA1 and BRCA2 ): sensitivity 0.94, specificity 0.51, PP 0.31, NP 0.97 | NA |
| 9 | Hughes et al. 2003 (A self-administered questionnaire) (24) | Breast and ovarian cancer | a) pedigree-oriented  b) patient-completed | Based on study criteria (No FH, insignificant FH, significant potentially high-risk) | Mammography, breast examinations, genetic counselling, genetic testing | *Clinical validity (*comparator: pedigree by surgical oncologist):  - 62.7% accuracy | *Identification of an increased risk:* - 9.4% had a significant family history (of those 62.7% were confirmed by the pedigrees) |
| 10 | Braithwaite et al. 2005 (GRACE) (25) | BC | a) pedigree-oriented  b) patient-completed | Based on Claus model using regional  Guidelines (low, moderate, high) | Breast awareness, use of mammography and genetic testing | NA | *Psychological impact:* - difference in risk perception and cancer related worries (between GRACE and nurse counselling: not statistically significant (P>0.05) for both outcomes |
| 11 | Emery et al. 2005; 2007 (GRAIDS) (26) | Breast, ovarian, colorectal, and endometrial cancers | a) pedigree-oriented  b) clinician-completed | Based on Claus model and Guidelines from the Regional Genetic Centre (average, moderate and high) | Further assessment and advice on screening for moderate and high risk;for high advice for genetic testing | NA | *Identification of an increased risk:* - more referrals than practise (MD= 3.2; CI 1.2–4.8; p=0.001); referrals more consistent with guidelines (OR 5.2; CI 1.7–15.8, p=0.006).  *Psychological impact:* - lower CWS scores after I (MD= 1.4; 95% CI 2.64 to 0.23, p=0.02); no differences in knowledge  *Other impact:* - the intervention increased GPs’ confidence in managing familial cancer |
| 12 | Ozanne et al. 2009; Williams 2012 (CRA Health ex. HughesRiskApp) (27) | Hereditary breast and ovarian cancer | a) pedigree-oriented  b) patient-completed | Based on BRCAPRO and Myriad (Ozanne) and EPA Framework (Williams); at high risk | Advice on attending GS and clinical decision support | NA | *Identification of an increased risk:*  *-* the tool has successfully identified 3.6% individuals eligible for genetic testing |
| 13 | Dekker et al. 2013 (Online referral test) (28) | Lynch syndrome | a) disease-oriented  b) patient- and clinician- completed | Based on the Dutch guidelines on hereditary  CRC; low, moderate and high | High risk - GS; moderate risk - surveillance colonoscopies | *Clinical validity* (comparator: pedigree)  - sensitivity was 91% for mutation carriers with CRC (n = 164) and 73% for all affected and non-affected mutation carriers (n = 420) | *Psychological impact:* 71% of 256 users reported that the referral test increased reassurance, certainty about their familial risk and/or certainty about referral. |
| 14 | Schultz et al. 2015 (Diagram and questionnaire-based web interfaces) (29) | Colorectal cancer | a) pedigree-oriented  b) patient-completed | Based on the New Zealand guidelines;  average, slightly increased, moderately increased and potentially high lifetime risk | Screening and surveillance of  patients with an increased risk of CRC | NA | NA |
| 1.2.2. PAPER-BASED | | | | | | | |
| 15 | Leggatt et al. 1993; Wallace et al. 2014 (FHQ) (30) | Breast and colorectal cancer | a) disease-oriented  b) patient-completed | Made by GPs following cancer genetic criteria (low, moderate, high) | Screening for those with an increased risk | *Clinical validity (comparator:* interview/telephone call with GS) - agreement for BC was 58.3%*;* for colon cancer was 80% | *Identification of an increased risk:* - 17% with increased risk |
| 16 | House et al. 1999; Rose et al. 2004 (FHQ) (31) | Colorectal cancer | a) disease-oriented  b) patient-completed | High risk using Amsterdam criteria; moderate and low risk using local guidelines | Screening for colonoscopy for high risk | *Clinical validity* (comparator: CC prevalence): *-* only 5% discrepancy in prevalence compared with NCRAS (172 vs 260 per 100.000 patients)  (comparator: GS review): - 5 inter-mediate risk patients were assigned to high risk group | *Identification of an increased risk:* - de novo identified in the high (20%) and moderate (39%) group; *Psychological impact:* - no difference observed in STAI and IES |
| 17 | Benjamin et al. 2003 (FCAT) (32) | Breast cancer | a) disease-oriented  b) patient-completed | Based on a broad agreement (low>16%, medium 16-25%, high<25%) | If positive, referral to a FH clinics or genetic department (more than 25%) | *Clinical validity* (comparator: interview with GS): - sensitivity was 92% (95% Cl, 84-97%), specificity was 83% (95% Cl, 54-94%); PPV 0.97 (95% Cl, 90-99%); NPV 0.68 (95% Cl, 45-68%) | NA |
| 18 | Hoskins et al. 2006; 2010 (PAT) (33) | Hereditary breast cancer | a) pedigree-oriented  b) clinician-completed | Based on the study’s scoring (potentially increased risk, high and low BRCA risk) | GS and referral for BRCA gene mutation analysis | *Clinical validity* (comprator: Frank model): Sensitivity 100%, specificity 93%, PPV 63%, NPV 100% | *Identification of an increased risk*: -18.9% of potentially increased risk; 2.2% of a high probability that BRCA mutation is present; The PAT did better than Gail model in identifying those at high risk) |
| 19 | MacLeod et al. 2007 (A 21-item FHQ) (34) | Inherited cardiovascular disease | a) pedigree-oriented  b) patient-completed | Based on the Scheuner et. al 1997 model (high, moderate, average) | No | NA | *Identification of an increased risk:* 64.1% of patients at high risk, 84% at moderate risk; 23% of questionnaires showed the same information as GPs notes |
| 20 | Murff et al. 2007 (FH form) (35) | Breast, ovarian and colorectal cancer | a) pedigree-oriented  b) patient-completed | Breast and ovarian based on the literature and CC based on the guidelines (increased risk) | No | NA | *Identification of an increased risk -* 6.2% subjects were screen as high risk; - more at-risk individuals identified: 29 vs 19 in the chart; more information on age as compared to the chart (81% vs 40% in the medical chart) |
| 21 | Ashton-Prolla et al. 2009 (FH-7 questionnaire) (36) | Breast cancer | a) disease-oriented  b) patient-completed | Based on ASCO criteria for increased risk | Positively answered to one question, referred for further assessment | Clinical validity (comparator: pedigree)  - sensitivity (87.6%) and specificity (56.4%)  - ICC for at least one positive answer was 0.84 | NA |
| 22 | Bellcross et al. 2009 (RST) (37) | Hereditary risk for breast/ovarian cancer | a) pedigree-oriented  b) patient-completed | Based on US Preventive Service Task Force (2005); positive high risk; negative, low risk | Referral for GS and testing for BRCA1/2 mutations | *Analytical validity* (comparator: randomly repeated RST): Concordance of 156/2464 was 96% (k = 0.75)  *Clinical validity (comparator:* pedigree by GS)  - sensitivity = 81.2%; specificity = 91.9%; discrimination accuracy = 0.87% | NA |
| 23 | Pieper et al. 2012 (38) | Colorectal cancer | a) pedigree-oriented  b) patient-completed | Based on the Network  against colorectal cancer guiideliness (one positive answer: increased risk) | NA | *Analytical validity (comparator:* an evaluation of an uptake of the questionnaire): - more positive responses on question 1 at t1 than at t3 (13% vs. 11%); - more “Don’t know” answers at t1 (t1: 19% vs. t2: 4%) in question 2 | *Identification of an increased risk:* - the tool identified 16% patients with increased risk; *Other impact:* - patients identified at increased risk for the first time had significantly less frequent physician contact (GP: 20% vs. 34%, gastroenterologist: 8% vs. 21%) |
| 24 | Niendorf et al. 2016 (39) | Hereditary cancer syndrome | a) disease-oriented  b) patient-completed  c) + telephone interview | Based on the Hampel Criteria; high, moderate and average risk | High risk received advice for GC, GC, list of local G services, and current guidelines | *Clinical validity* (comparator: GC):  - agrmt for an increased risk was 87% (n=500) | Identification: - 66.4% were found to be at high risk, 20.4% at moderate and 13.2% at average risk for hereditary cancer syndrome |
| 25 | Koné et al. 2018 (A 4-item questionnaire) (40) | Colorectal cancer | a) pedigree-oriented  b) clinician-completed | No | No | *Analytical validity (*comparator: general practitioners' responses): *-* good agrmt on Q1 (first-relative diagnosed with CC): kappa 0.82; Cl 0.58-1.10)*;* - Q 2-4 could not be validated with this methodology | NA |
| 26 | Mariani et al. 2020 (STELO) (41) | Inherited cancer  syndromes | a) pedigree-oriented  b) patient-completed | Scientific literature and disease guidelines; increased risk | One positive answer, send to the GS | *Clinical validity (comparator: clinical records):*  - 76.5% agrmt; sensitivity 88.5%, specificity 52.3% | NA |
| 2. PERSONAL USE TOOLS | | | | | | | |
| 1.1. GENERIC/MULTIFACTORIAL | | | | | | | |
| 1.1.1. COMPUTERIZED/ WEB BASED | | | | | | | |
| 27 | Cohn et al. 2010 (Health Heritage©) (42) | 87 diseases | a) pedigree-oriented  b) patient-completed  c) web-based | Based through thorough disease specialist framework (high, moderate, avarage) | Based on the level of risk, includes screening, treatment and prevention | *Clinical validity* (comparator: pedigree with GS)  - identification of patients at risk for disease: 60% vs 24% by usual care; in 17/25 conditions HH was more sensitive than usual care | *Identification of increased risk:* - 42% of the participants discovered new health risks*; Behavioural change: -* 56% indicated the risk would change their health behaviour |
| 1 | Yoon et al., 2009, Rubinsten et al. 2011; Ruffin et al. 2011; O’Neill et al. 2009 (Family Healthware™) (16) | Colon, breast and ovarian cancer, CHD, diabetes and stroke | a) pedigree-oriented  b) patient-completed | Based on the predefined framework (low, moderate, high) | Based on various factors (level of risk, sex, age, health behaviours, e.g. diet), lifestyle changes and screening | *Clinical validity* (comparator*:* studies with comparable risk-stratification); good agreement (CHD: 30%-35% vs 32%-33%; diabetes: 8%-12% vs 7%-16%) | *Identification of an increased risk:* - 34% moderate/high risk for cancer; *Behaviour change:* 3% eating healthier and 4% increase in PA after personalized risk-tailored vs age- and sex- specific messages |
| 28 | Facio et al. 2010; Owens et al. 2011; Feero et al. 2015  (My Family Health Portrait) (43) | 6 diseases | a) pedigree-oriented  b) patient-completed | Algorithm based on the accepted guideliness (weak, moderate, strong) | Based on the level of risk (screening, genetic testing, treatment options); advice to contact the physician | *Clinical validity* (pedigree by GS): - sensitivity = 67–100%; specificity = 92–100%*;* agrmt: 94–99% for diabetes and colon, breast, and ovarian cancer; 58% for coronary artery disease; 83% for stroke  - (for CC only): sensitivity 81%, specificity 90%; PPV 48%; NPV 98%; agrmt of 0.54 (k) | NA |
| 29 | Sweet et al. 2015; Thomas et al. 2019 (Family Healthlink) (44) | Coronary heart disease, cancer | a) pedigree-oriented  b) patient-completed | Algorithms developed from published literature (high, moderate, average) | Risk surveillance and risk reduction (moderate and high); GS only for high risk | *NA* | *Identification of an increased risk:* - high risk criteria for cancer was found in 22.2%; 24.3%, for CHD, and 10.4% for both diseases; 91.5% of the assignments were confirmed; of these 32% underwent genetic testing and 40.5% of these had found a mutation |
| 2 | Wang et al. 2015; Diez et al. 2019 (VICKY) (17) | Various cancers, heart disease, diabetes, hypertension, stroke | a) pedigree-oriented  b) patient-completed | No | Patient receives a pedigree chart (PDF), which can be used for healthcare  providers | *Analytical validity (comparator: MFHP)*  - VICKY identified 86% of FDR, and 42% of SDR; (accuracy for both: 55%)  *Clinical validity* (comparator: pedigree with GS and compared to MFHP): VICKY identified a greater number of health conditions overall (49% VICKY vs 31% MFHP, p=.008); hypertension (47% vs 15%, p=.001) and type 2 diabetes (54% vs 22%; p=.004) | NA |
| 2.2. SINGLE DISEASE | | | | | | | |
| 2.2.1. COMPUTERIZED/ WEB BASED | | | | | | | |
| 30 | Welch et al. 2015; 2020 (ItRunsInMyFamily) (45) | Various cancers | a) disease-oriented  b) patient-completed | Yes (not specified) | Yes (not specified) | *NA* | Identification: More than 80% of our respondents reported having a family member with cancer. |
| 3. CANCER (SCREENING) CLINICS TOOLS | | | | | | | |
| 3.1. GENERIC/MULTIFACTORIAL | | | | | | | |
| 3.2. COMPUTERIZED/ WEB BASED | | | | | | | |
| 31 | Doerr et al. 2014 (MyLegacy/ MyFamily) (46) | Cancers, diabetes, abdominal aortic aneurysm; | a) pedigree-oriented  b) patient-completed  c) EMR | Based on guidliness on specific disease (high and avarage) | GS to high-risk patients via telemedicine | NA | NA |
| 29 | Sweet et al. 2015; Thomas et al. 2019 (Family Healthlink) (44) | Coronary heart disease, cancer | a) pedigree-oriented  b) patient-completed | Algorithms developed from published literature (high, moderate, average) | Risk surveillance and risk reduction (moderate and high); GS only for high risk | *NA* | *Identification of an increased risk:* - high risk criteria for cancer was found in 22.2%; 24.3%, for CHD, and 10.4% for both diseases; 91.5% were confirmed; of these 32% underwent genetic testing and 40.5% had mutation |
| 3.2. SINGLE DISEASE | | | | | | | |
| 3.2.1. COMPUTERIZED/ WEB BASED | | | | | | | |
| 32 | Westman et al. 2000; Sweet et al. 2002; Kelly et al. 2008 (Jameslink) (47) | 27 types of cancer | a) disease-oriented  b) patient-completed | Based on NCCN hereditary cancer criteria  guidelines and literature sources | Advice on life-style and attending GS | NA | *Identification* *of an increased risk* - I identified 27.9% of high-risk*;* medical records failed to identified patients 6.9/14%); *Psychological impact:* - 42% changed their perceptions; *Behavioural change:* - 20% made behavioural changes); 53.8% intended to speak to GP about their risk |
| 33 | Kallenberg et al. 2015; 2018 (48) | Familial and hereditary colorectal cancer | a) disease-oriented  b) patient-completed | Based on Dutch nationwide referral criteria for Lynch  syndrome or FCC | An automated referral GS for LS and surveillance colonoscopies for FCC | *Clinical validity (*comparator: pedigree interview by GC): Sensitivity was 100% *(95%CL* 63–100) and specificity was 97 % (95 % CI 91–99 %); IOA: 100 % in referral decisions | NA |
| 34 | Guivatchian et al. 2017 (49) | Colorectal cancer | a) pedigree-oriented  b) patient-completed | Based on Bethesda criteria, expert opinion and National guidelines; | GS considered (increased) and GS recommended (high risk) | NA | *Identification of an increased risk:* - the tool identified 10% and 9% patients as high risk for CRC in cohorts 1 and 2, respectively; - of 69 high-risk subjects, 33% underwent genetic evaluations and 10% carried germline mutations associated with cancer predisposition |
| 3.2.2. PAPER-BASED | | | | | | | |
| 17 | Benjamin et al. 2003 (FCAT) (32) | Breast cancer | a) disease-oriented  b) patient-completed | Based on a broad agreement (low>16%, medium 16-25%, high<25%) | If positive, referral to a FH clinics or genetic department (more than 25%) | *Clinical validity* (comparator: interview with GS): - sensitivity was 92% (95% Cl, 84-97%), specificity was 83% (95% Cl, 54-94%); PPV 0.97 (95% Cl, 90-99%); NPV 0.68 (95% Cl, 45-68%) | NA |
| 35 | Fisher et al. 2003 (Questions about BC in your family) (50) | Breast cancer | a) disease-oriented  b) patient-completed | No | No | *Clinical validity* (comparator: interview with GS)*:* - 100% agreement with risk assessment after interview by GS | NA |
| 36 | Cohn et al. 2008 (Are you at risk for hereditary breast cancer?) (51) | Inherited breast and ovarian cancer | a) disease-oriented  b) patient-completed | One or more risk factors mean increased risk (based on team's experience, past work) | Advising to further GS for increased risk | NA | *Identification of an increased risk:* - 15% of participants recognized that they are at risk |
| 37 | Vogel et al. 2012 (52) | Lynch syndrome  or hereditary BC and OC | a) pedigree-oriented  b) patient-completed | USPSTF,  NCCN and SGO guidelines; at risk | Referral to GS | Clinical validity (comparator: interview with GS and electronic medical record): - 81% concordance with genetic interview; - 62% increase in referrals by questionnaire as compared to EMR | *NA* |
| 38 | Wood et al. 2014 (53) | Breast or colon cancer | a) disease-oriented  b) clinician-completed | Based on specific guidliness (ASCO, NCCN, US Preventative Services Task Force) | Referral for GS | *NA* | Identification of an increased risk: - 25.6% patients with BC or CRC were referred for genetic counselling/testing |
| 39 | Schiavi et al. 2015 (SCGS Questionnaire) (54) | Sarcoma | a) pedigree-oriented  b) patient-completed | LFS or LFL criteria (did or did not report a  FH of LF syndrome–type cancers) | Referral to the genetic clinics | *NA* | Identification of an increased risk: - FH of cancer (as far as TRD) was reported 69% |
| 40 | Campacci et al. 2017 (PSQ) (55) | Breast, ovarian, and colorectal cancer | a) pedigree-oriented  b) patient-completed | Various disease specific criteria included; one positive response mean at-risk | Referred to the GC | *Clinical validity* (comparator: pedigree and telephone call by a nurse with an experience in cancer genetics); -sensitivity (94%) and specificity (75%), a kappa of 0.64 | Identification: - 15.6% were at-risk |
| 24 | Niendorf et al. 2016 (39) | Hereditary cancer syndrome | a) disease-oriented  b) patient-completed  c) + telephone interview | Based on the Hampel Criteria; high, moderate and average risk | High risk received advice for GC, list of local G services, guidelines and explanation of GS | *Clinical validity* (comparator: GC):  - agrmt for an increased risk was 87% (n=500) | Identification: - 66.4% were found to be at high risk, 20.4% at moderate and 13.2% at average risk for hereditary cancer syndrome |
| 34 | Guivatchian et al. 2017 (49) | Colorectal cancer | a) pedigree-oriented  b) patient-completed | Based on Bethesda criteria, expert opinion and National guidelines; | GS considered (increased) and GS recommended (high risk) | NA | *Identification of an increased risk:* - the tool identified 10% and 9% patients as high risk for CRC in cohorts 1 and 2, respectively; - of 69 high-risk subjects, 33% underwent genetic evaluations and 10% carried germline mutations associated with cancer predisposition |
| 4. GENETIC CLINICS TOOL | | | | | | | |
| 4.1. GENERIC/MULTIFACTORIAL | | | | | | | |
| 4.1.1. PAPER-BASED | | | | | | | |
| 41 | Cole et al. 1978 (Genetic FH questionnaire) (56) | Various | a) pedigree-oriented  b) clinician-completed | No | No | *Analytical validity* (comparator: final pedigree obtained from revision of answered responses): - half of the pedigrees required changes or additional information | NA |
| 42 | Bensen et al. 1999 (FHQ) (57) | CHD, diabetes, hypertension and asthma | a) pedigree-oriented  b) patient-completed | NA | NA | *Analytical validity* (comparator: relative’s self-reported disease status) - sensitivity (CHD = 81%; diabetes = 56%, asthma = 39%); most specificity score were above 90% | NA |
| 4.2. SINGLE DISEASES | | | | | | | |
| 4.2.1. COMPUTERIZED/WEB BASED | | | | | | | |
| 43 | Acheson et al. 2006 (GREAT) (58) | 24 types of cancer | a) pedigree-oriented  b) patient-completed | High, low BRCA probability and potentially increased risk; criteria not listed | Yes, but not specified | *Analytical validity (comparator: paired pedigree):* - 94% of FDRs, 67% of SDRs, and 38% of TDRs;-retest reliability (Ø 0.94 for FDR and Ø 0.91 in SDR.  *Clinical validity* (comparator: interview with GS)*:* - agrmt (κ =0.70) for BC, acceptable agrmt (κ =0.54) for CC, and good for other cancers (κ =0.62) | NA |
| 12 | Ozanne et al. 2009; Williams 2012 (CRA Health ex. HughesRiskApp) (27) | Hereditary breast and ovarian cancer | a) pedigree-oriented  b) patient-completed | Based on BRCAPRO and Myriad (Ozanne) and EPA Framework (Williams); at high risk | Advice on attending GS and clinical decision support | NA | *Identification of an increased risk:*  *-* the tool has successfully identified 3.6% individuals eligible for genetic testing |
| 4.2.2. PAPER-BASED | | | | | | | |
| 44 | Armel et al. 2009 (59) | Breast and ovarian cancer | a) pedigree-oriented  b) patient-completed | No | No | Analytical validity (comparator: pedigree with GS): - after interview 12% families needed to change their pedigree, this led to revised probability estimate for having a BRCA1/2 mutation | NA |
| 5. INTERNAL MEDICINE | | | | | | | |
| 5.1. GENERIC/MULTIFACTORIAL | | | | | | | |
| 5.1.1. PAPER-BASED | | | | | | | |
| 45 | Frezzo et al. 2003 (FHQ) (60) | Cardiovascular disease cancers, tromboses | a) pedigree-oriented  b) patient-completed | Based on review, consensus statements, and current guidelines (high, moderate, low) | Referral for GS or genetic testing* | NA | *Identification of an increased risk:* - higher identification of high risk as compared to the chart review (61.5% vs 39.7%) |

Abbreviations. NA – not assessed. FH – family history. OR – odds ratio. GP – general practitioner. MD – mean difference. CWS – cancer worry scale. GS – genetic screening. GC – genetic counselor. BC – breast cancer. CC – colorectal cancer. CHD – coronary heart disease. CRC – IHD - ischaemic heart disease. MFHP - My Family Health Portrait. PA – physical activity. agrmt – agreement. PPV – positive predictive valua. NPV – negative predictive value. RST – referral screening tool. WICKY – Virtual Counsellor for Knowing Your Family History. RAG – Risk Assessment in Genetics. FHAT – Family History Assessment Tool. GRACE – Genetic Risk in the Clinical Environment. GRAIDS – Genetic Risk Assessment in an Intranet and Decision Support. CRA health – cumulative risk assessment health. FCAT – Familial Cancer Assessment Tool. PAT – Pedigree Assessment Tool. RST – Referral Screening Tool. STELO – Sindromi dei Tumori Ereditati Lynch e Ovaio/mammella. K – kappa. ROC curve – receiver operating characteristics. ICC – interclass correlation coefficient.
