# Supplementary material for: Family history tools for primary care: A systematic review
Source: Eur J Gen Pract. 2022 May 5;28(1):75–86. doi: 10.1080/13814788.2022.2061457 (PMC9090347; doi:10.1080/13814788.2022.2061457)
Supplement: Supplemental Appendix 2 [file IGEN_A_2061457_SM7904.docx]

Appendix 2. PubMed Search Strategy

| 1 | Pedigree |
| --- | --- |
| 2 | Genetic testing |
| 3 | Genetic predisposition to disease |
| 4 | 2 OR 3 |
| 5 | Medical history taking |
| 6 | Medical records systems |
| 7 | Computerized |
| 8 | Electronic health records |
| 9 | Decision support techniques |
| 10 | Decision making |
| 11 | Computer-assisted |
| 12 | 5 OR 6 OR 7 OR 8 OR 9 OR 10 OR 11 |
| 13 | 1 AND 4 AND 12 |
